# Secretome analysis of breast cancer cells to identify potential target proteins of *Ipomoea turpethum* extract-loaded nanoparticles in the tumor microenvironment

**DOI:** 10.3389/fcell.2023.1247632

**Published:** 2023-10-12

**Authors:** Sanskriti Swami, Mohd Mughees, Irengbam Rocky Mangangcha, Sana Kauser, Saima Wajid

**Affiliations:** ^1^ Department of Biotechnology, School of Chemical and Life Sciences, Jamia Hamdard, New Delhi, India; ^2^ Department of Zoology, Deshbandhu College, University of Delhi, New Delhi, India

**Keywords:** breast cancer, nano LCMS/MS, secretome analysis, *Ipomoea turpethum*, nanoparticles, bioinformatics

## Abstract

**Background:** Breast cancer is the leading cause of frequent malignancy and morbidity among women across the globe, with an increment of 0.5% incidences every year. The deleterious effects of traditional treatment on off-target surrounding cells make it difficult to win the battle against breast cancer. Hence, an advancement in the therapeutic approach is crucial. Nanotechnology is one of the emerging methods for precise, targeted, and efficient drug delivery in cells. The previous study has demonstrated the cytotoxic effect of *Ipomoea turpethum* extract on breast cancer cells delivered via NIPAAM-VP-AA nanoparticles (NVA-IT). Manipulating the tumor microenvironment (TME) to inhibit cancer progression, invasion, and metastasis seems to be very insightful for researchers these days. With the help of secretome analysis of breast cancer cells after treatment with NVA-IT, we have tried to find out the possible TME manipulation achieved to favor a better prognosis of the disease.

**Method:** MCF-7 and MDA MB-231 cells were treated with the IC_50_ value of NVA-IT, and the medium was separated from the cells after 24 h of the treatment. Nano LCMS/MS analysis was performed to identify the secretory proteins in the media. Further bioinformatics tools like GENT2, GSCA, GeneCodis 4, and STRING were used to identify the key proteins and their interactions.

**Result:** From the nano LCMS/MS analysis, 70 differentially expressed secretory proteins in MCF-7 and 191 in MDA MB-231 were identified in the cell’s media. Fifteen key target proteins were filtered using bioinformatics analysis, and the interaction of proteins involved in vesicular trafficking, cell cycle checkpoints, and oxidative stress-related proteins was prominent.

**Conclusion:** This study concluded that *I. turpethum* extract-loaded NIPAAM-VP-AA nanoparticles alter the secretory proteins constituting the TME to cease cancer cell growth and metastasis.

## 1 Introduction

Cancer is one of the leading causes of death globally, with approximately 10 million deaths reported in 2020, among which most newly diagnosed cases were of breast cancer. The number of breast cancer cases increased by 2.26 million in 2020, contributing to 11.7 percent of all new cancer incidences. According to the cancer statistics stated by the ACS 2022, out of all the newly estimated cancer cases among women in the United States, 31% would be breast cancer, which has shown an increment in incidence rates by 0.5% per year since the mid-2000s (cancer statistics, 2022—Siege—2022—CA: A Cancer Journal for Clinicians—Wiley Online Library, n.d.). In 2020 alone, 685,000 deaths were reported, making it one of the leading causes of frequent malignancy and morbidity among women across the globe (IARC, 2023). Breast cancer is a diverse condition characterized by aberrant cell development in the inner lining of mammary ducts or lobules, which emerges from a series of sequential modifications/mutations altering the functioning of various cell types. Different types of breast cancer have been listed based on the morphological arrangement of cells, the extent of invasion, and the frequency of occurrence. The behavior and subtype of breast cancer affect the treatment strategy (Maughan et al., 2010). Reportedly, there are primarily three cancer treatment approaches: surgery (such as mastectomy and lumpectomy), chemotherapy, and radiotherapy. Perhaps, chemotherapy is the most often employed to cure different types of cancer (Waks and Winer, 2019). However, there are challenges associated with conventional drug delivery methods like lack of selective toxicity and poor penetration (due to the presence of barriers across the transportation), resulting in a short half-life and poor effectiveness (Giger et al., 2013). High radiation exposure, deleterious off-target damaging effects on the surrounding areas, and financial stress due to expensive surgical treatments are some of the other limitations of the traditional treatment strategy. Approximately, an expenditure of $29 billion was estimated in 2020 alone for the breast cancer treatment of the US population, implying the financial burden faced by cancer patients (Institute of Medicine (US) and National Research Council (US) Committee on the Early Detection of Breast Cancer Statistics, 2001; Levenson, 2007). Nanotechnology, as a fast-evolving area, offers exciting possibilities for early detection and therapy of human cancer. Nanoparticle-based formulations bypass biological barriers, allowing for longer blood circulation times, simultaneous tumor targeting, and increased drug accumulation in tumors (Liyanage et al., 2019; Zubair et al., 2020). They have a higher therapeutic efficiency than traditional treatment due to high specificity, effective drug targeting, and no solubility and stability issues. In the previous study, it was demonstrated that *Ipomoea turpethum* extract-loaded polymeric NIPAAM-VP-AA nanoparticle (NVA-IT) induces cytotoxicity in breast cancer cell lines (MCF-7 and MDA MB-231) by regulating pathways involved in apoptosis and cell proliferation (Mughees et al., 2019). *I. turpethum* is a perennial plant known for its medicinal benefits since ages in ailments like fever, edema, asthma, hemorrhoids, dysplasia, flatulence, gout, paralysis, abdominal tumors, and worm infestation (Gupta and Ved, 2017; Hamedi et al., 2017). Recent studies have also reported the cytotoxic effect of the plant extract on cancer cells (Arora et al., 2017; Mughees and Wajid, 2020). The current understanding of breast cancer implies that other than neoplastic cells, the tumor microenvironment (TME) also plays a very important role in the progression and metastasis (Mughees et al., 2021). The TME consists of cells like macrophages, lymphocytes, extracellular matrix, and secretory components from proliferating cells. Many researchers are focusing on targeting the TME for prognostic, diagnostic, and therapeutic purposes (Xue et al., 2008; Croucher et al., 2016; Luque et al., 2021). In the present study, we have analyzed the effect of *I. turpethum* extract-loaded NIPAAM-VP-AA nanoparticle treatment on the TME via secretory proteins present in the medium of the tumor cells. Nano LCMS/MS analysis was performed to identify the differentially expressed secretory proteins present in the media of cell lines (MDA MB-231 and MCF-7).

## 2 Materials and methods

### 2.1 Chemicals

N-isopropyl acrylamide (NIPAAM, Acros Organics (United States) was recrystallized with N-hexane (distilled) at 40°C and was vacuum-dried). N-vinyl 2- pyrrolidone (VP) and acrylic acid (AA) were purchased from Acros Organics (United States) and freshly distilled before use. N, N′-Methylenebisacrylamide (MBA), ferrous ammonium sulfate (FAS) (anhydrous), and ammonium persulfate (APS) were purchased from Sigma-Aldrich (St. Louis, United States). Dulbecco’s modified Eagle medium (DMEM), fetal bovine serum (FBS), trypsin (with 0.5% EDTA), streptomycin (100X), potassium chloride, disodium hydrogen phosphate, sodium chloride, and potassium dihydrogen phosphate were purchased from HiMedia (United States). MCF-7 and MDA-MB 231 cell lines were procured from the National Centre for Cell Science (NCCS), Pune, India. *I. turpethum* was procured from the FRLHT (Foundation of Revitalization of Local Health Traditions, Bangalore, India) and further confirmed by the expert Dr. Sunita Garg (Head, Raw Materials Herbarium & Museum, CSIR-NISCAIR, New Delhi, India) with the reference number NISCAIR/RHMD/Consult/2015/2826/19.

### 2.2 Synthesis of *Ipomoea turpethum* extract-loaded nanoparticles

*I. turpethum* root extract-loaded NIPAAM-VP-AA nanoparticles were synthesized with the method discussed by Mughees et al. (2019), Mughees et al. (2019). Briefly, NIPAAM, VP, and AA were allowed to copolymerize with a free radical reaction in the presence of FAS and APS as activators and initiators respectively in the nitrogen gas environment. After the successful polymerization of nanoparticles, the ethanolic root extract of the *I. turpethum* plant was loaded in its core with the physical entrapment method.

### 2.3 Cell culture

Breast cancer cell lines MCF-7 and MDA MB-231 were procured from the National Centre for Cell Science (NCCS), Pune, Maharashtra, India. Dulbecco’s modified Eagle medium (DMEM) with 10% fetal bovine serum and 100X penicillin was used for culturing the cells at 37°C, 95% air, and 5% CO_2_.

### 2.4 Treatment with NVA-IT

After the cells attained a confluency of 95%, they were treated with the dose of IC_50_ value, which has been calculated previously (Mughees et al., 2019) i.e., 221.82 μg/mL and 171.13 μg/mL for MCF-7 and MDA MB-231, respectively. After 24 h of the treatment, the media and the cells were separated with centrifugation for 10 min at 400 g.

### 2.5 Secretome analysis

The media collected after the treatment of cells with NVA-IT were subjected to secretory protein analysis using nano LCMS/MS.

#### 2.5.1 Sample preparation

The samples were dissolved in 6M Gn-HCl (50 mM TRIS, pH 8.8) buffer. A protein sample (50 ug) was used for digestion and reduced with 5 mM TCEP (tris(2-carboxyethyl) phosphine). Alkylation was performed with 50 mM iodoacetamide and further digested with trypsin (1:50, trypsin/lysate ratio) for 16 h at 37°C. The resultant digests were cleaned using a C18 silica cartridge to remove the salt and dried using a speed vacuum. The dried pellet was resuspended in buffer A (2% acetonitrile and 0.1% formic acid).

#### 2.5.2. Mass spectrometric analysis of peptide mixtures

Mass spectrometric analysis of peptide mixtures was performed using the RSLC Nano system (Thermo Fisher Scientific) coupled to Thermo Fisher-QExactive Plus equipped with the nano electrospray ion source. One microgram of the sample was loaded on a 50-cm C18 column and 3.0 μm EASY-Spray column (Thermo Fisher Scientific). Peptides were eluted with a 0%–40% gradient of buffer B (80% acetonitrile and 0.1% formic acid) at a flow rate of 300 nL/min and injected for MS analysis. Liquid chromatography gradients were run for 100 mins. MS1 spectra were acquired in the Orbitrap at 70K resolution. Dynamic exclusion was employed for 10 s excluding all charge states for a given precursor. The MS2 spectra were acquired at 17,500 resolutions.

#### 2.5.3 Data processing

All the samples once processed were subjected to a mass spectrometry run. Raw files containing mass/charge values were generated for each of the samples. The mass spectrometry proteomics data have been deposited to the ProteomeXchange Consortium via the PRIDE (Perez-Riverol et al., 2022) partner repository with the dataset identifier PXD042366. Furthermore, these raw files were analyzed using Thermo Proteome Discoverer (v2.2) against the UniProt Human database. For SEQUEST and Amanda search, the precursor and fragment mass tolerance were set at 10 ppm and 0.5 Da, respectively. The protease used to generate peptides, i.e., enzyme specificity was set for trypsin/P (cleavage at the C terminus of “K/R: unless followed by “P”) along with the maximum missed cleavage value of 2. Carbamidomethyl on cysteine as fixed modification, oxidation of methionine, and acetylation of the N terminus were considered variable modifications for database search. Both peptide spectrum match and protein false discovery rate (FDR) were set to 0.01 FDR.

### 2.6 Bioinformatics analysis

#### 2.6.1 Classification of proteins using PANTHER analysis

The differentially expressed proteins (DEPs) in MCF-7 and MDA MB-231 were classified using Gene Ontology analysis and the Protein Analysis Through Evolutionary Relationships (PANTHER 17.0) classification system (Mi et al., 2021; PANTHER, 2023). Proteins were clustered into five categories, namely, molecular function, protein class, biological process, pathways, and cellular components.

#### 2.6.2 Expression and survival analysis

To assess the alteration of gene expression of the proteins, their level of expression in normal and tumor tissue was studied using online bioinformatics tools. The DEPs were subjected to tissue-wide gene expression patterns across normal and tumor breast tissue using the Gene Expression database of Normal and Tumor tissues 2 (GENT2) (Park et al., 2019). The expression of a gene in the different subtypes was also obtained by GENT2. To evaluate the correlation between the gene expression and overall survival of breast cancer patients, the survival analysis was performed using GENT2 and KM plotter (1,246 patients). A *p*-value < 0.05 was considered statistically significant.

#### 2.6.3 Immune cell infiltration and drug sensitivity

The correlation between the expression of individual genes and infiltration of 24 immune cells was calculated in TCGA-BRCA (breast invasive carcinoma) samples by using the Gene Set Cancer Analysis (GSCA) online tool (Liu et al., 2023). The association of mRNA expression and 24 immune cell infiltrates was estimated through Spearman correlation analysis by Immune Cell Abundance Identifier (ImmuCellAI) (Miao et al., 2020) in GSCA. The calculated GSVA score represents the level of integration of gene set expression that is positively correlated with gene set expression. The interdependence of mRNA expression and drug sensitivity was estimated using Genomics of Drug Sensitivity in Cancer (GDSC) and Genomics of Therapeutics Response Portal (CTRP) data. Pearson correlation analysis was performed to obtain the correlation between gene mRNA expression and drug IC_50_. The *p*-value was adjusted by the FDR. The top 30 drugs were plotted against the gene expression using GSCA.

#### 2.6.4 Functional enrichment analysis

Functional enrichment analysis was executed by the online bioinformatics tool GeneCodis 4 (gene annotations co-occurrence discovery) which provides singular and modular enrichment analysis (SEA and MEA) (Garcia-Moreno et al., 2022). Using this tool, KEGG pathways, Reactome, PharmGKB, DisGeNET, and Gene Ontology cellular component analyses of the selected genes have been performed. Finally, to discover the interactions and associations (both known and predicted) among the selected proteins, Cytoscape and STRING 11.5 online tools were used (Szklarczyk et al., 2021).

### 2.7 Statistical analysis

The statistical analysis was performed by using GraphPad Prism software version 8.0 to find the statistical significance of differentially expressed secretory proteins between untreated and NVA-IT-treated breast cancer cells (MCF-7 and MDA MB-231) (Mavrevski et al., 2018). An unpaired *t*-test was applied to compare the two groups, and the *p*-value was <0.05. The association of mRNA expression and 24 immune cell infiltrates using Spearman correlation analysis and the correlation between gene mRNA expression and drug IC_50_ were estimated using Pearson correlation analysis. The *p*-value was adjusted by the FDR.

## 3 Result

### 3.1 Nano LCMS/MS

**FIGURE 1:**
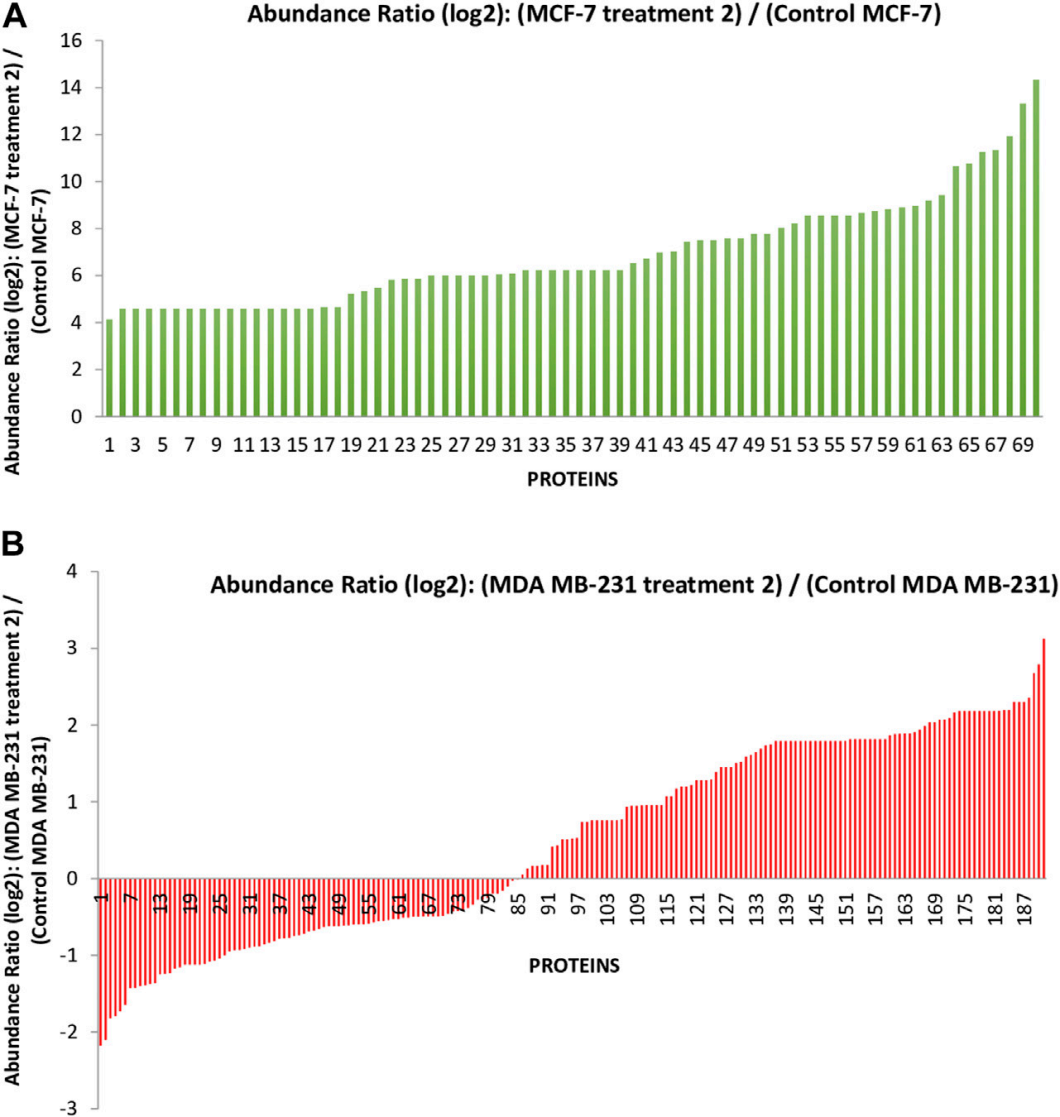
The graph represents the differentially expressed proteins present in the secretome of **(A)** the MCF-7 cell and **(B)** MDA MB-231 after treatment with *Ipomoea turpethum* extract-loaded nanoparticles identified by nano LCMS/MS.

In the previous study, we reported that the *I. turpethum* extract-loaded NIPAAM-VP-AA nanoparticles (NVA-IT) cause cytotoxicity in breast cancer cell lines (MCF-7 and MDA MB-231). Proteomic analysis of the cellular proteins demonstrated that there were many apoptotic and cell cycle arrest pathways involved in the process. Currently, we are determined to identify the altered secretory proteins present in the medium, which represents the changes in the TME of the cancerous cells. Through nano LCMS/MS analysis of the MCF-7 and MDA MB-231 cell’s medium after treatment with the IC_50_ value of NVA-IT, we identified a total of 358 secretory proteins in the former and 261 secretory proteins in the latter cell line. The untreated cell’s secretome accounted for 71 and 180 proteins in MCF-7 and MDA MB-231 cells, respectively. Out of the total proteins identified, 191 in MDA MB-231 and 70 in MCF-7 cells were significantly dysregulated compared to untreated cells (*p* < 0.05) (Figure 1, Supplementary Tables S1, S2).

### 3.2 PANTHER analysis

Most of the targeted proteins in the MCF-7 cell secretome were involved in the molecular function like binding (elongation factor 1-alpha1 and heterogeneous nuclear ribonucleoprotein C-like 2) and catalytic activity (trypsin-1, peroxisomal bifunctional enzyme, and nucleoside diphosphate kinase A). PANTHER GO-slim biological process categorization associated proteins in the cellular processes like cellular component organization and biogenesis, cell death, cell cycle, cellular metabolism (tubulin alpha-1B chain, Fas-binding factor 1, gelsolin, cytochrome C, and tubulin alpha-1B chain), localization (lactotransferrin and vitamin D-binding protein), developmental process (thrombospondin-1), and metabolic processes. Pathways like cytoskeletal regulation by the Rho GTPase, Wnt signaling pathway, cadherin signaling pathway, gonadotrophin-releasing hormone pathway, inflammation mediated by the chemokine and cytokine signaling pathways, and integrin signaling pathway cover most of the target proteins in the PANTHER GO-slim pathway analysis. The protein class classification showed that maximum proteins belonged to the cytoskeletal protein class, protein binding activity modulator, and RNA metabolism protein. These DEPs , for the most part, produce the intracellular anatomical structure, organelles, and cytoplasmic structure, revealed by the cellular component PANTHER GO-slim (Supplementary Figure S2).

In MDA MB-231, pathway analysis also showed the involvement of proteins in glycolysis, blood coagulation, EGF signaling pathway, FGF signaling pathway, and Huntington and Parkinson disease-related pathways, along with those present in MCF-7. Biological process analysis revealed the involvement of biological regulation in protein, cellular, and metabolic processes. Metabolite interconversion enzyme, transfer and carrier proteins, and chaperon protein classes were mainly targeted by the NVA-IT treatment in MDA MB-231 (Supplementary Figure S3).

### 3.3 Expression and survival analysis

**FIGURE 2:**
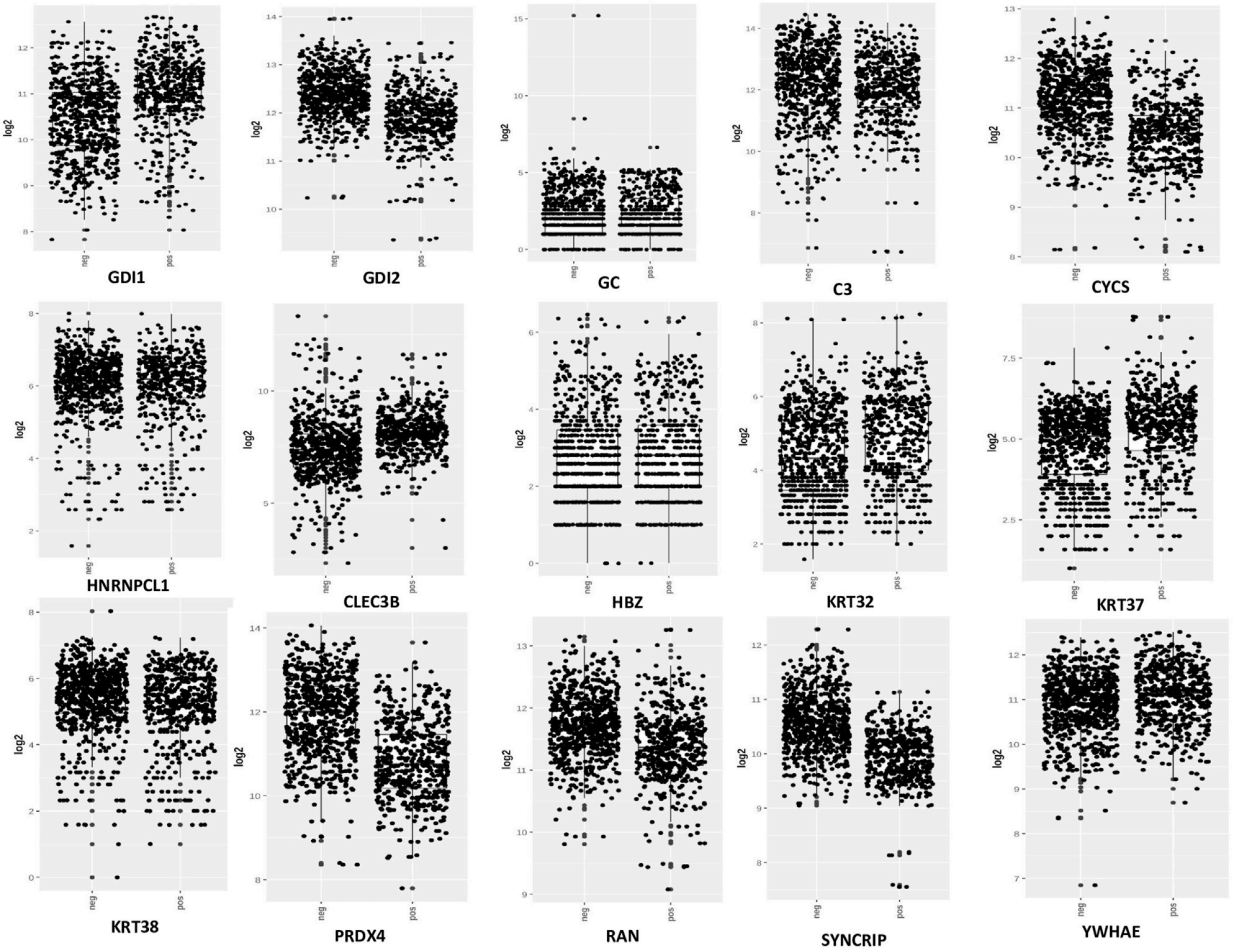
Expression analysis of selected proteins in ER+ and ER−breast cancer patients (GENT2 tool).

**FIGURE 3:**
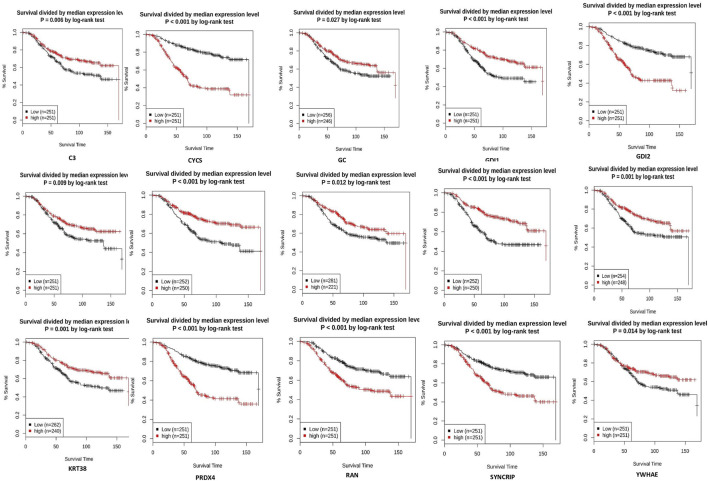
Survival analysis of selected proteins in breast cancer patients (GENT2 tool).

From the expression and survival analysis, 15 proteins have been sorted out (Table 1). Proteins that showed counter expression after NVA-IT treatment than their expression in cancer patient data (GENT2) were selected and further excluded based on the ER levels (Figure 2). Proteins that correlated with better prognosis based on a survival curve (*p*-value < 0.05) have been selected for further evaluation (Figure 3). Proteins like complement C3 (C3), vitamin D-binding protein (GC), heterogeneous nuclear ribonucleoprotein C-like 1(HNRNPCL1), keratin, type I cuticular Ha2 (KRT32), keratin, type I cuticular Ha7 (KRT37), keratin, type I cuticular Ha8 (KRT38), hemoglobin subunit zeta (HBZ), and tetranectin (CLEC3B) were upregulated, and GTP-binding nuclear protein Ran (RAN), Rab GDP dissociation inhibitor alpha (GDI1), Rab GDP dissociation inhibitor beta (GDI2), cytochrome c (CYCS), 14-3-3 protein epsilon (YWHAE), heterogeneous nuclear ribonucleoprotein Q (SYNCRIP), and peroxiredoxin-4(PRDX4) were downregulated after NVA-IT treatment in MCF-7 and MDA MB-231 cell lines. Proteins like HNRNPCL1 and HBZ, CLEC3B, KRT32, and PRDX4*.*, were exclusively expressed in MCF-7 and MDA MB-231, respectively whereas C3, CYCS, GC, GDI1, and GDI2 were expressed in both cell lines (Supplementary Table S1). MCF-7 and MDA MB-231 represent different subtypes of breast cancer, i.e., luminal A and triple-negative breast cancer (TNBC); hence, the expression of selected proteins was also analyzed in different subtypes of breast cancer (Supplementary Figure S4).

**TABLE 1:** Gene expression profile across cancer experiments [GPL570 platform (HG-U133_Plus_2)] and cell lines after NVA-IT treatment.

S.no	Gene symbol	Fold change in breast cancer tissue (GENT2 expression analysis)	*p*-value (GENT2)	The expression after treatment with NVA-IT (nano LCMS/MS)	Targeted cell line (nano LCMS/MS)
1	C3	−0.599	<0.001	Upregulated	MCF-7
2	CYCS	0.304	<0.001	Downregulated	MDA MB-231
3	GC	−0.457	<0.001	Upregulated	MCF-7
4	GDI1	0.165	<0.001	Downregulated	MDA MB-231
5	GDI2	0.202	<0.001	Downregulated	MDA MB-231
6	HNRNPCL1	−0.133	0.007	Upregulated	MCF-7
7	HBZ	−0.199	0.001	Upregulated	MDA MB-231
8	CLEC3B	−2.199	<0.001	Upregulated	MDA MB-231
9	KRT32	−0.509	<0.001	Upregulated	MDA MB-231
10	KRT37	−0.249	<0.001	Upregulated	MDA MB-231
11	KRT38	−0.638	<0.001	Upregulated	MDA MB-231
12	PRDX4	0.287	<0.001	Downregulated	MDA MB-231
13	RAN	0.211	<0.001	Downregulated	MDA MB-231
14	SYNCRIP	0.097	<0.001	Downregulated	MDA MB-231
15	YWHAE	0.123	0.021	Downregulated	MDA MB-231

### 3.4 Immune cell infiltrates and drug sensitivity

**FIGURE 4:**
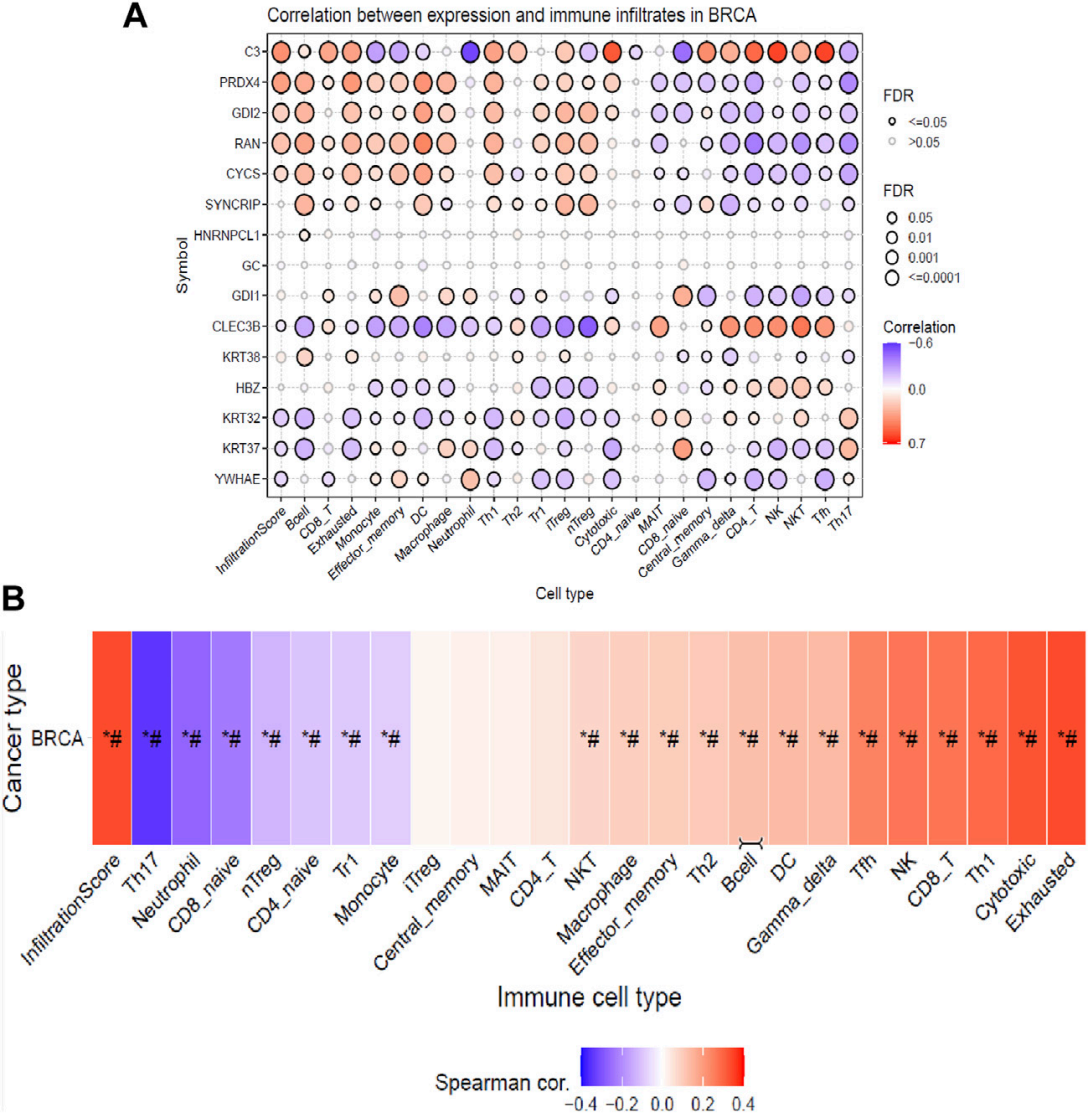
Immune cell infiltration analysis of the gene set by the ImmuCellAI tool representing **(A)** the correlation between the expression of individual genes and immune infiltrates in breast cancer and **(B)** heat map showing the correlation of the gene set with the immune cell type.

**FIGURE 5:**
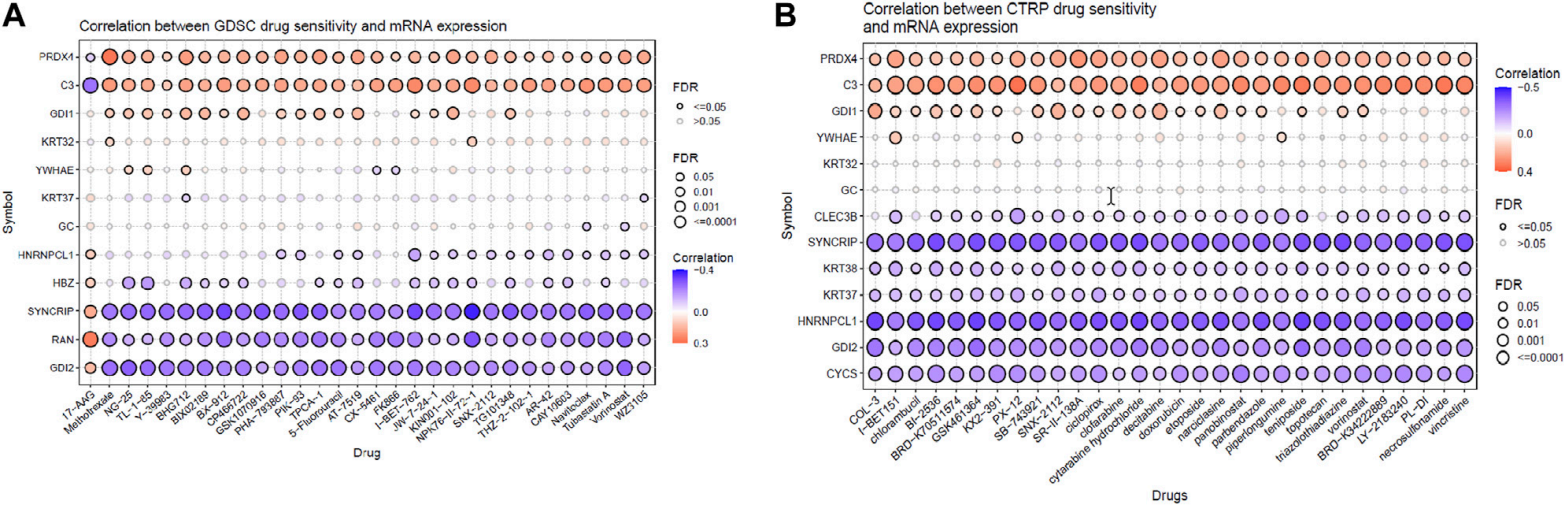
Pharmacogenomic studies of the gene set by **(A)** GSCA and **(B)** CTRP, representing the drug sensitivity of target genes toward 30 pan-cancer drugs.

From the previous analysis from GENT2, 15 key proteins were selected for GSCA analysis for immunogenomic and pharmacogenomic studies. The immune cell infiltration analysis, by the ImmuCellAI tool, showed that the selected genes are positively correlated with the immune cells like exhausted, cytotoxic T cells, CD8 T, natural killer cells, T helper cell type 1, and T follicular helper cells. T helper type 17, neutrophils, CD8 naïve T cells, and natural regulatory T cells were found to be negatively correlated with the set of the selected genes. The scrutiny of pharmacogenomics by GSCA screened 30 drugs that showed sensitivity/resistance in the presence of the gene set. In the bubble plot, red nodes represent positive correlations, blue nodes represent negative correlations, and larger nodes indicate stronger correlations. A positive correlation signifies that high expression of the gene favors drug resistance and *vice versa*. PRDX4, GDI1, and C3 showed a positive correlation with most of the drugs in both GDSC and CTRP, whereas SYNCRIP and GDI2 showed a negative correlation toward almost all the drugs (Figures 4, 5).

### 3.5 Functional enrichment analysis

**FIGURE 6:**
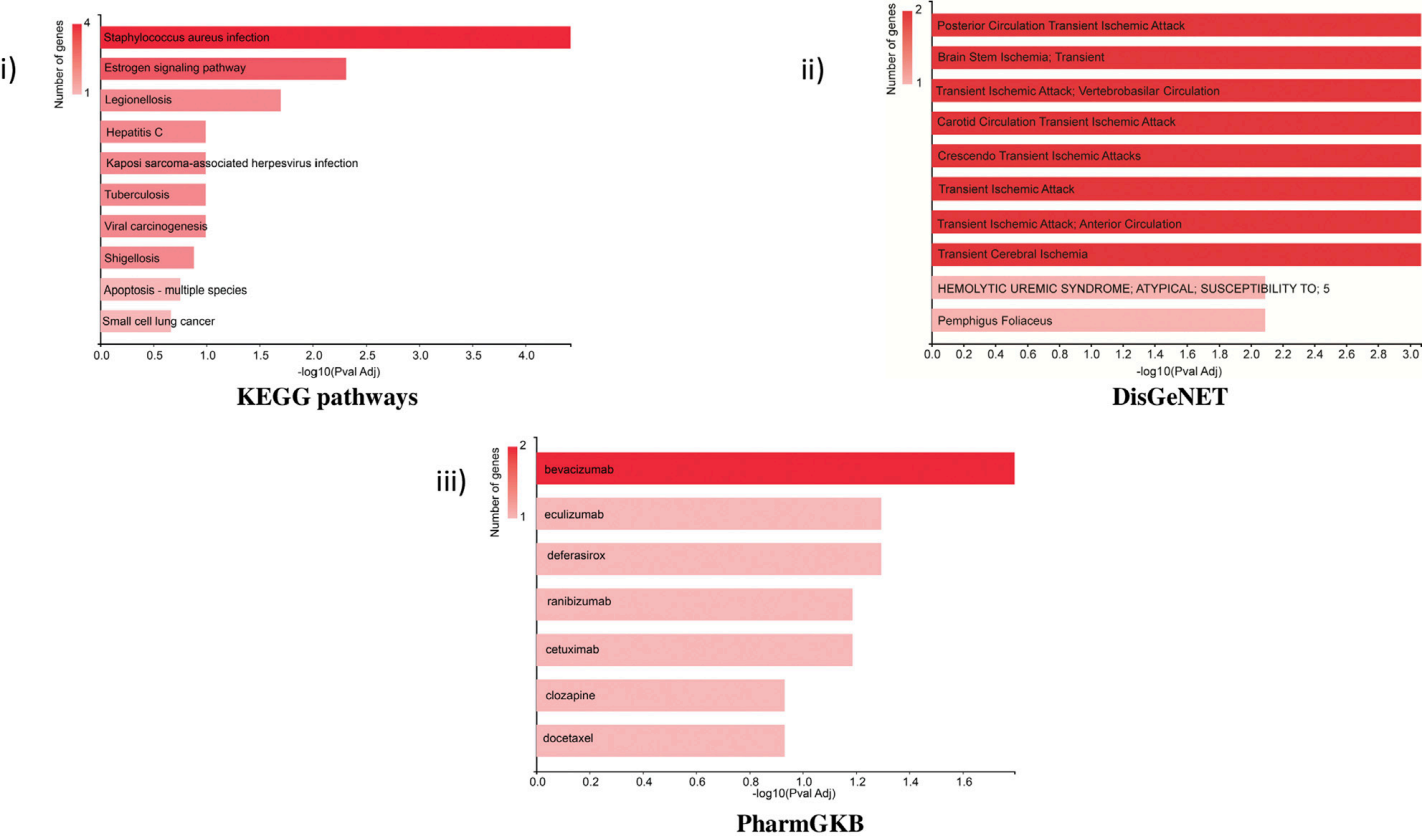
Functional enrichment analysis of selected proteins by GeneCodis representing i) KEGG pathway, ii) DisGeNET, and iii) PharmGKB related to the set of selected genes.

KEGG pathway: The bar chart of the KEGG pathways related to the set of genes showed that most genes were involved in *Staphylococcus aureus* infection (KRT32, C3, KRT38, and KRT37) and the estrogen signaling pathway (KRT32, KRT38, and KRT37). The estrogen signaling pathway is one of the key targeted pathways in the NVA-IT treatment in the MDA MB-231 cell line (ER-negative cell line). Other proteins involved in cancer-related pathways like hepatitis C, viral carcinogenesis, Kaposi sarcoma-associated herpesvirus infection, and some apoptosis-related proteins were also targeted by the treatment. The PharmGKB bar chart displayed seven drugs, out of which five are potent anti-cancer agents. Targets of bevacizumab, cetuximab, ranibizumab, docetaxel, and clozapine were found in PharmGKB analysis. DisGeNET analysis informed about the other diseases, which can be a potential target for treatment. Apoptosis pathway, complement activation, and molecular trafficking-related proteins were found to be most prominent in the Reactome pathway analysis (Supplementary Figure S1). Cellular component analysis of the proteins revealed that most of the proteins make the part of extracellular exosomes, cytosol, intermediate filament, and secretory granule lumen. The RNA nuclear export complex, apoptosome, GAIT complex, and CRD-mediated mRNA stability complex had the highest relative enrichment (Figure 6).

### 3.6 Interactome analysis by the STRING online tool

**FIGURE 7:**
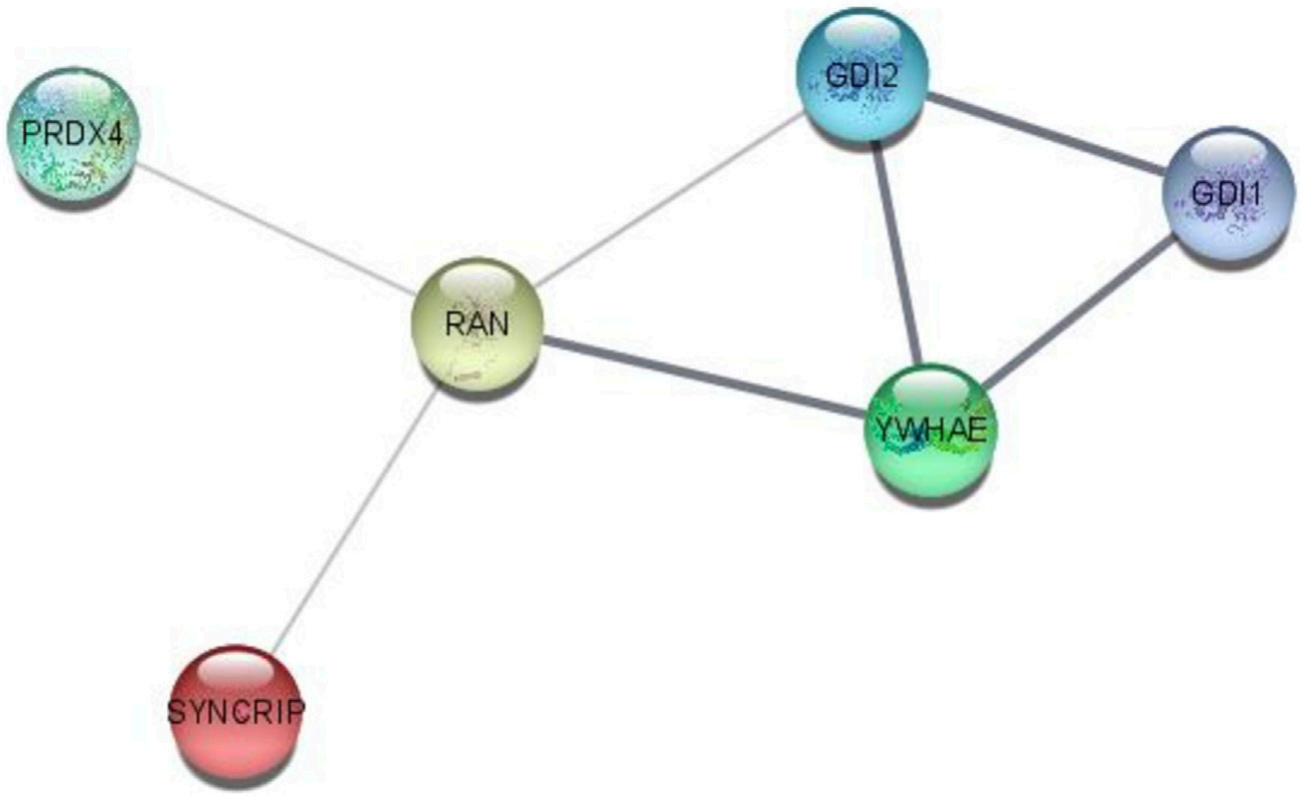
STRING interactome analysis to find the interaction of selected targeted proteins after NVA-IT treatment.

Interactome analysis of 15 selected distinguished proteins showed direct interaction of six proteins, i.e., GDI1, GDI2, YWHAE, RAN, PRDX4, and SYNCRIP. Vitamin D-binding protein and complement C3 were also interlinked directly with each other. The interaction among GDI1, GDI2, PRDX4, YWHAE, RAN, and SYNCRIP is well-documented either from a curated database or determined experimentally. In the interactome study, the interplay of proteins involved in apoptosis and survival pathways and Rab GDP dissociation inhibitor activity was prominent (Figure 7).

## 4 Discussion

The conjugation of nanoparticles with herbal medicine is one of the most promising tools for targeting cancer, according to recent studies. In the current investigation, we have used NIPAAM-VP-AA polymeric nanoparticles for targeting cancer due to their sensitivity to pH and temperature (Ahmad et al., 2013). This double-triggered nature of nanoparticles makes them release the drug in the proximity of the TME only. The cytotoxic nature of NVA-IT toward cancer cells (MCF-7 and MDA MB-231) has been reported in our previous research (Mughees et al., 2019). In this study, further secretome analysis has been achieved to observe the effect of NVA-IT on the TME (Croucher et al., 2016). After treatment with the IC_50_ dose of NVA-IT (24 h post-treatment), the medium of the cells was collected and subjected to nano LCMS/MS analysis. The total proteins found through nano LCMS/MS in NVA-IT-treated MCF-7 and MDA MB-231 secretome were compared to those in untreated cells. In the case of MCF-7 cells, 70 differentially expressed proteins were found to be upregulated with fold change >4, in which proteins involved in vesicle-mediated trafficking, apoptosis, necroptosis, and cell proliferation were noteworthy. Some proteins involved in the regulatory pathways like the TGF-beta signaling pathway, p53 signaling pathway, PI3K-Akt signaling pathway, the Hippo signaling pathway, and Wnt signaling pathways were also found distinguished after NVA-IT treatment. In MDA MB-231 cells, 84 proteins were significantly downregulated and 106 were significantly upregulated (*p*-value <0.05). Proteins involved in cell migration, keratinocyte migration, and chondrocyte proliferation were found to be downregulated. Proteins involved in the regulation of cell growth through interactions with the ECM and cytokines were also downregulated, for example, SPARC, SPARC-like protein-1, collagen alpha-1(I) chain, and fibronectin (Insua-Rodríguez and Oskarsson, 2016). Other than apoptosis and mitophagy, proteins involved in pathways like the estrogen signaling pathway, glycolysis/gluconeogenesis, pyruvate metabolism, and HIF1 signaling pathway were also affected after treatment.

Bioinformatics plays a very important role in analyzing the potential functional and structural characteristics of a gene or protein molecule. Here, the classification of the molecular functions and pathways of differentially expressed proteins (DEPs) in MCF-7 and MDA MB-231 cells after NVA-IT treatment was performed using PANTHER (Mi et al., 2021). The analysis showed significant enrichments of the extracellular binding proteins; proteins with catalytic activity like hydrolase activity, transferase activity, lyase activity, and oxidoreductase activity; structural molecules; and enzyme regulator activity proteins (Figures 2, 3). Since, altered extracellular binding proteins are known to modulate the TME, facilitating tumor invasion and migration (Insua-Rodríguez and Oskarsson, 2016; Gleissner et al., 2019), proteins like thrombospondin-1 (THBS1), which is a cytokine, and extracellular matrix proteins like EGF-containing fibulin-like extracellular matrix protein 1 (EFEMP1), fibulin-1 (FBLN1), and fibromodulin (FMOD) have well-known roles in tumor metastasis and drug resistance.

MCF-7 and MDA MB-231 represent two different subtypes of breast cancer, i.e., luminal A and TNBC, respectively. Therefore, the effect of NVA-IT would be different in both cell lines (Jia et al., 2014). PANTHER functional/pathway classification analyses of the DEPs showed that there were different pathways and functional proteins in the two cell types. Biological processes like the cellular response to stimuli, protein folding, cell communication, and signal transduction proteins were significantly enriched in NVA-IT-treated TNBC cells. Pathways involved in Parkinson’s disease, blood coagulation, glycolysis, EGF receptor signaling pathway, FGF signaling pathway, plasminogen-activating cascade, and PI3K signaling pathway were found to be specific to MDA MB-231, whereas the integrin signaling pathway and apoptosis signaling pathway were enriched in both MCF-7 and MDA MB-231 cell types, but more prominent in the latter. Similarly, chaperons and transfer/carrier proteins along with scaffold/adapter proteins, extracellular matrix proteins, and defense/immunity proteins were more enriched in MDA MB-231 than in MCF-7 cells. Therefore, cellular functional and pathway enrichment analyses of the NVA-IT-treated breast cancer cells showed that the treatment had a major impact on the proteins involved in external encapsulating structure, envelope, and sarcomere-related proteins in TNBC cells.

Further investigations on the proteins using GENT2 (Park et al., 2019), which provides the gene expression database of normal and tumor tissue samples across 72 different tissues, revealed that NVA-IT treatment had a major impact on the expressions of the dysregulated genes in breast cancer. In NVA-IT treatment, 62 proteins were exclusively differentially expressed in MDA MB-231 cells, two proteins were exclusively differentially expressed in MCF-7 cells, and 28 proteins were differentially expressed in both cell types. Heterogeneous nuclear ribonucleoprotein C-like 1(HNRNPCL1) upregulated in MCF-7 neutralizes basic proteins, consequently involved in nucleosome assembly and a potential biomarker in cancer (Chen et al., 2016; Gao et al., 2020). STRING interaction analysis showed the direct interaction of vitamin D-binding protein (GC) and complement C3 (C3) targeted in MCF-7. Vitamin D-binding protein-derived macrophage-activating factor induces apoptosis and phagocytosis in breast cancer cells (Lopes et al., 2012; Thyer et al., 2013), whereas C3 is the foremost player in innate immune response manipulated in conventional chemotherapy (Michlmayr et al., 2010; Bauer et al., 2019; Shu et al., 2020). Complement C3 known for facilitating metastasis is downregulated in the counterpart TNBC MDA MB-231 cell line, suggesting that NVA-IT uniquely targets the same protein in different subtypes. Similarly, cytochrome C (CYCS), a well-known apoptosis player, is highly upregulated in MCF-7 (fold change 5) but downregulated in MDA MB-231 (Jiang and Wang, 2004a; Pessoa, 2022).

MCF-7 and MDA MB-231 represent the two different phenotypes of breast cancer based on the expression of estrogen receptors, i.e., ER+ and ER−, respectively (Alghanem et al., 2020). Based on this scenario, 15 proteins were selected based on their ER status and better prognostic value. The expression of selected proteins also varied in different breast cancer subtypes, i.e., luminal, luminal A, luminal B, HER2, TNBC, and basal (Figure 6). The expression analysis of DEPs revealed that the proteins with better prognosis like HNRNPCL1, CLEC3B, HBZ, KRT32, KRT37, KRT38, C3, GC, GDI1, and YWHAZ are upregulated, and the proteins with poor prognosis like CYCS, GDI2, PRDX4, RAN, and SYNCRIP are found to be downregulated post NVA-IT treatment (*p*-value < 0.05, Figure 5). Keratin, type I cytoskeletal 17 (KRT17), heat shock-related 70 kDa protein 2 (HSPA2), and keratin, type I cytoskeletal 14 (KRT14) have lower expressions, whereas complement factor B (CFB) and Rab GDP dissociation inhibitor alpha (GDI1) have higher expressions in breast cancer patients than in normal healthy controls, but on NVA-IT treatment, these proteins showed reversed expressions in MDA MB-231 cells. Inhibition of GDI1 by NVA-IT treatment was observed only in TNBC MDA MB-231 cells, but not in MCF-7 cells where its higher expression was not affected by the treatment. Furthermore, from the survival analysis, we also found that well-documented prognostic markers, i.e., keratin, type I cytoskeletal 17 (KRT17), heat shock-related 70 kDa protein 2 (HSPA2), complement factor B (CFB), and keratin, type I cytoskeletal 14 (KRT14), showed altered expression after NVA-IT treatment. They show a significant impact on overall survival (OS) and disease-free survival (DFS) among breast cancer patients. This implies that NVA-IT might have a more pernicious impact on metastatic TNBC breast cancer cells. A total of 28 proteins were found to be altered after NVA-IT treatment in both cell lines (Supplementary Table S3). Based on expression analysis, survival analysis, and ER status, we selected five proteins, i.e., CYCS, GDI1, GDI2, C3, and KRT2, for further bioinformatics analysis. CYCS (cytochrome C) is a known apoptotic marker that induces intrinsic apoptotic pathways and helps in the amplification of extrinsic apoptotic pathways (Jiang and Wang, 2004a). GDP dissociation inhibitor (GDI) proteins contribute to cellular processes like vesicular trafficking, cytoskeletal organization signal transduction, and cell proliferation (Wang and Wen, 2022). GDI1 is a prognostic marker, and its high expression is unfavorable in breast cancer treatment. Complement C3 (C3) protein is a crucial part of the innate immune response’s complementary system. The association of complement C3 expression with breast cancer primary tumor and lymph node metastatic breast cancer has been documented by Popeda et al., pointing toward targeting C3 for therapeutic purposes (Popeda et al., 2021). Vitamin D-binding protein (GC) facilitates the immune system response against cancer cells by proper activation of macrophages (Gc-MAF), leading to their phagocytosis (Rozmus et al., 2020). Studies have shown that Gc-MAF is an advanced and promising immunotherapy for the treatment of cancer. However, FDA approval has not been granted for now (Saburi et al., 2017).

Infiltration of different immune cells is the characteristic of different cancer types, and investigation of the immune cell infiltration sheds more light on the progression of the tumor (Barnes and Amir, 2017; Steven and Seliger, 2018). Therefore, further analysis of the correlation between gene expressions of the DEPs with the infiltration of different immune cells in breast cancer patients (BRCA) is profitable. The analysis indicated that based on their cumulative infiltration scores, immune cells like T17, neutrophils, CD8_naive, and nTreg, which have poor prognoses in breast cancer, were found to have negative correlations with the gene expressions of the DEPs. This could mean their potential suppression by the expression of DEPs (Mouchemore et al., 2018; Sadeghalvad et al., 2021; Moallemi-Rad et al., 2023). On the other hand, infiltration of immune cells with a good prognosis like CD8 T cells (CD8_T), natural killer (NK), T helper type 1(TH1), T follicular helper cells (TfH), exhausted T cells (exhausted), and cytotoxic T cells was positively correlated with the expressions of DEPs potentially enhancing their infiltrations (Zhao et al., 2019; Sadeghalvad et al., 2021). Moreover, natural regulatory T cells (nTreg), CD4 naïve T cells (CD4_naive), type 1 regulatory T cells (Tr1), and monocytes were negatively correlated, whereas natural killer T cells (NKT) , macrophages, effector memory cells, T helper type 2 (TH2), B cells, dendritic cells (DCs), and gamma delta T cells (gamma delta) positively correlated with the gene expressions of DEPs, exhibiting their extensive impact on immune infiltrations in breast cancer patients. The literature has shown that breast cancer patients have elevated blood nTreg, Tr1, and effector/memory (EM) nTregs levels and correlate with poor clinical outcomes (Ostapchuk et al., 2018). An increased level of EM nTreg cells was also reported to be associated with an increased risk of recurrence and metastasis. Monocyte expansion is one of the key characteristics of the TME, which favors tumor progression which is dependent upon different cytokines, breast cancer stages, and subtypes (Amer et al., 2022). Monocytes proliferate actively in TNBC but not in luminal A breast cancer subtypes, and they can differentiate into macrophages which can be either pro-inflammatory (M1), supporting the proliferation of NK cells and cytotoxic cells, or it can be anti-inflammatory (M2) by suppressing the immune cell proliferation (Amer et al., 2022). These effects are partially associated with increasing CD8^+^ T cells and CD4^+^ T cell infiltration in the TME and promoting Th1/Th2 balance toward Th1 response (Zhao et al., 2019). The TME consists of both immune suppressor and activating cells, which vary among different cancer types. The account of different types of immune cell infiltrates will predict the better prognosis of the disease. After NVA-IT treatment, there is a significant enhancement in the immune cells that predict better outcomes in the patients, such as T cells like CD8, T helper type 1, T follicular helper cells, exhausted T cells, cytotoxic T cells, natural killer T cells, macrophages, T helper type, and B cells. On the other hand, infiltration of poor prognostic cells like neutrophils, CD8 naïve T cells, natural Treg, and effector/memory T cells is reduced with the expression of DEPs. Thus, the findings indicated that expressions of DEPs enhanced infiltration of cancer-targeting immune cells in the breast TME, and the NVA-IT treatment could potentially provide a better prognosis.

In addition, the pharmacogenomic study of the DEPs in GSCA also showed that the downregulation of proteins such as PRDX4, GDI1, and YWHAE and the upregulation of proteins such as CLEC3B, KRT38, KRT37, and HNRNPCL1 were linked to higher sensitivity toward 30 pan-cancer CTRP drugs. Proteins such as C3, SYNCRIP, CYCS, and GDI2 were linked to insensitivity or resistance toward CTRP drugs. In the case of GDSE drugs, mRNA expression of PRDX4, GDI1, HNRNPCL1, and HBZ provides sensitivity to the top 29 drugs, whereas C3, KRT32, YWHAE, SYNCRIP, RAN, and GDI2 showed lower sensitivity/insensitivity or resistance to these drugs. Pharmacogenomic studies of the genes using PharmGKB in GeneCodis gave the highest enrichment to bevacizumab, eculizumab, and cetuximab. Bevacizumab inhibits angiogenesis by targeting the VEGF signaling pathway and improving immune response (Bear et al., 2012; Tiainen et al., 2016; Boucher et al., 2021). Eculizumab is used to treat chemotherapy-related thrombotic microangiopathy, and deferasirox is an iron-chelating agent which might be used in conjugation with chemotherapy to reduce the recurrence of cancer in TNBC. Cetuximab is a monoclonal antibody targeting epidermal growth factor receptor (EGFR) for inhibiting the EGF signaling pathway in breast cancer (Zhang et al., 2019). Ranibizumab is antiangiogenic and binds to VEGF alpha. Clozapine is an antipsychotic drug, and it also inhibits cell proliferation by inducing autophagic cell death in two non-small-cell lung carcinoma cell lines (Yin et al., 2015). Thus, these target genes of NVA-IT treatment in breast cancer cells could also be sensitive to the existing pan-cancer chemotherapy drugs, which could provide a better prognosis (Jia et al., 2022).

The cellular components related to the gene set feature extracellular exosomes, secretory granules, and cytosol which were functionally and structurally related to the TME of the cells (Wolf-Dennen and Kleinerman, 2020). Identification of potential pathways and functions associated with the selected target proteins, using KEGG in GeneCodis, showed the highest enrichment of the estrogen signaling pathway, hepatitis C, and apoptosis pathway. This showed that the highest number of genes among the DEPs were involved in the estrogen signaling pathway, which is the most important function of breast cancer manifestation (Suba, 2014; Rashmi et al., 2019; Cheng et al., 2022). Reactome pathway analyses of the genes for their molecular pathways also showed the intrinsic pathway for apoptosis, SMAC (DIABLO) binding to IAPs, activation of caspases through apoptosome-mediated cleavage, and release of apoptotic factors from the mitochondria pathways as the highest enriched molecular functions and pathways among the genes. Formation of the cornified cell envelope, Rab regulation of trafficking, RAB GEFs exchanging GTP for GDP on RABs, keratinization, alternative complement activation, and TP53 regulating metabolic genes were other important enriched pathways. Disease association studies of the genes using DisGeNET showed an association of the genes with ischemia related to the brain and heart, for example, brain stem ischemia, carotid circulation transient ischemic attack, and transient cerebral ischemia, which indicates toward NVA-IT drug repurposing and understanding drug action and adverse effect mechanisms in other diseases (Piñero et al., 2017).

The STRING interactome analysis showed the direct interaction of six out of fifteen proteins. These proteins are the key players in vesicular trafficking (GDI1, GDI2, and RAN), oxidative stress regulation (PRDX4), and cell cycle checkpoints (YWHAE) (Jia et al., 2019; Mei et al., 2020; Bolado-Carrancio et al., 2021; El-Tanani et al., 2023). HnRNPs are known for their roles in RNA processing like alternate splicing, HnRNA packing, and mRNA stability. Knockdown of HnRNP Q led to decreased cell proliferation in cancer cells, making it an efficient target of anti-cancer drugs (Yoo et al., 2009). GTP-binding nuclear protein Ran is not only involved in nucleo-cytoplasmic transport of macromolecules over the nuclear envelope but also in mitotic spindle formation and nuclear assembly post mitosis, which makes it an influential cancer therapy target. Downregulation of RAN post-NVA-IT treatment signifies the occurrence of cell cycle arrest and cell death in cancer cells (El-Tanani et al., 2023). PRDX4 is the only secreted form of peroxiredoxin in the cell responsible for accurate nascent protein folding and avoiding any oxidative stress-related misfolding. However, its overexpression has been proven to facilitate tumor initiation and progression, drug resistance, and reoccurrence (Jia et al., 2019). During the cell cycle, 14-3-3 regulates DNA damage monitoring points by MAPK activation and regulates the integrin signaling pathway, maintains cytoskeleton dynamics, and promotes cell growth (Hermeking and Benzinger, 2006). In addition, 14-3-3 epsilon is found upregulated in breast cancer cells, and its knockdown decreases Snail and Twist transcription factor expression which is responsible for epithelial–mesenchymal transition (Hodgkinson et al., 2012; Zhang et al., 2015; Mei et al., 2020). A similar outcome has been observed by NVA-IT treatment in the MDA MB-231 cell line for all the interacting proteins. To the best of our knowledge, MCF-7 is non-invasive and less aggressive than MDA MB-231, which is invasive in nature. In this study, we have shown that NVA-IT induces cell death and inhibits cell proliferation in the non-invasive, ER-dependent cancer cells, whereas it inhibits invasion, metastasis, and angiogenesis in the aggressive type of cancer cells. NVA-IT regulates many components of the TME responsible for cancer progression, migration, and aggressiveness.

## 5 Conclusion

In conclusion, the differential secretome analysis of MCF-7 and MDA MB-231 cell lines treated with *Ipomoea turpethum* extract-loaded polymeric nanoparticles deduced that NVA-IT induces cytotoxicity in cancer cells not only by internal pathways but also by affecting the TME. The secretome analysis of cancer cells post NVA-IT treatment showed that many proteins like thrombospondin-1, fibronectin, and vitronectin involved in cancer progression, metastasis, and angiogenesis have been affected. The interactome analysis demonstrates the interaction of molecular trafficking and microtubule assembly proteins like PRDX4, GDI1, GDI2, and YWHAE, targeted by NVA-IT. NVA-IT treatment manipulates the TME by enhancing the flow of anti-tumor immune cells, affecting immunity booster pathways, and increasing the flow of cytokines against cancer cells, making the TME unfit for diseased cells. Further *in vivo* analysis and clinical study of this herbal-based nanoformulation are required for analyzing its therapeutic efficacy.

## Data Availability

The datasets presented in this study can be found in online repositories. The names of the repository/repositories and accession number(s) can be found below: http://www.ebi.ac.uk/pride, PXD042366.
