# Supplementary material for: Secretome analysis of breast cancer cells to identify potential target proteins of Ipomoea turpethum extract-loaded nanoparticles in the tumor microenvironment
Source: Front Cell Dev Biol. 2023 Oct 12;11:1247632. doi: 10.3389/fcell.2023.1247632 (PMC10602817; doi:10.3389/fcell.2023.1247632)
Supplement: Supplementary file 1 [file Presentation1.pdf]

Figure S1: GeneCodis analysis of selected proteins representing. i) Reactome Pathway, and iii) Gene Ontology Cellular Component related to the set of selected genes.

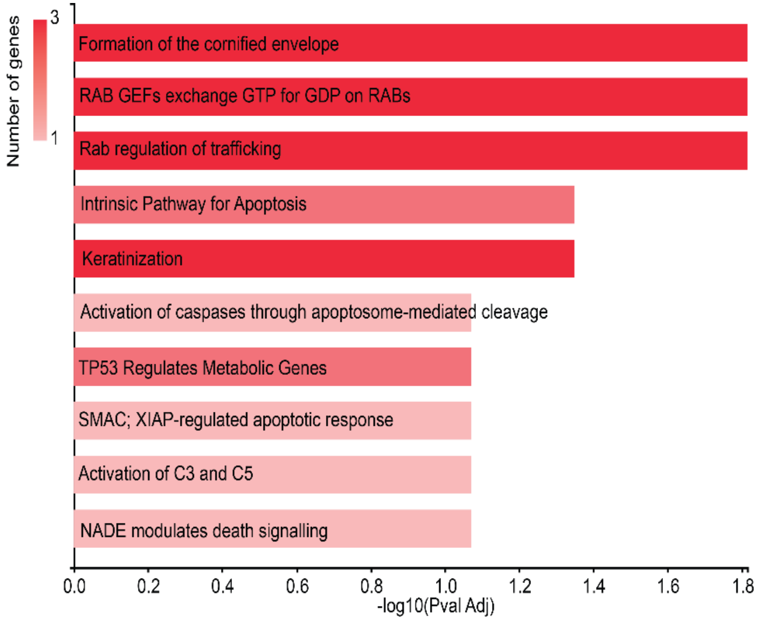

Reactome Pathways

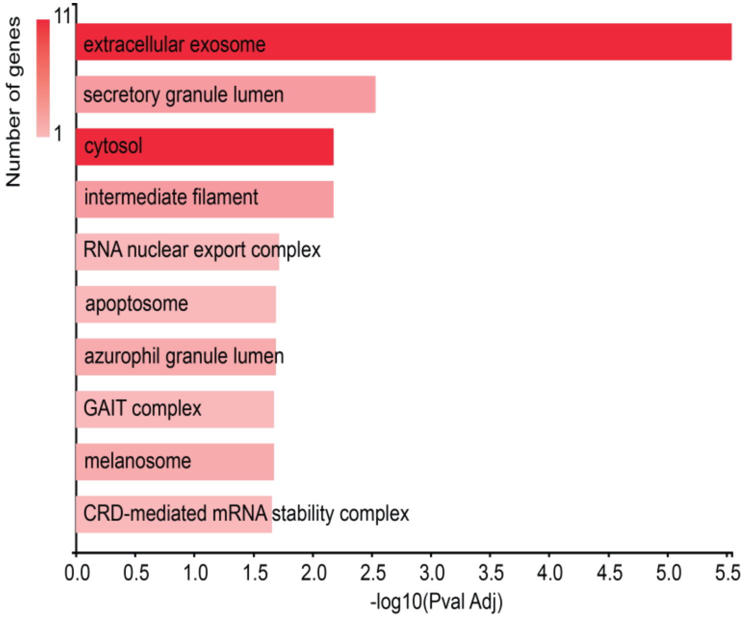

Gene Ontology Cellular Component

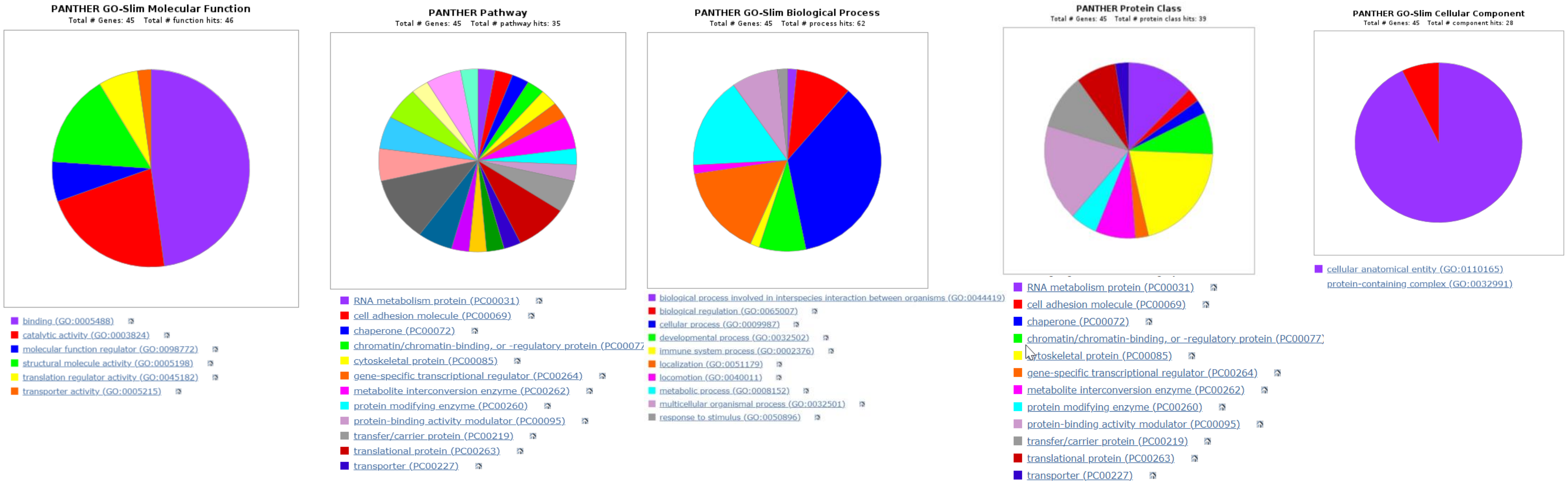

Figure S2 Pie charts showing differentially expressed secretome proteins of MCF-7 after treatment with NVA-IT, involved in pathways, biological processes, cellular components, and molecular functions. The values are represented as mean± SEM(n=3).

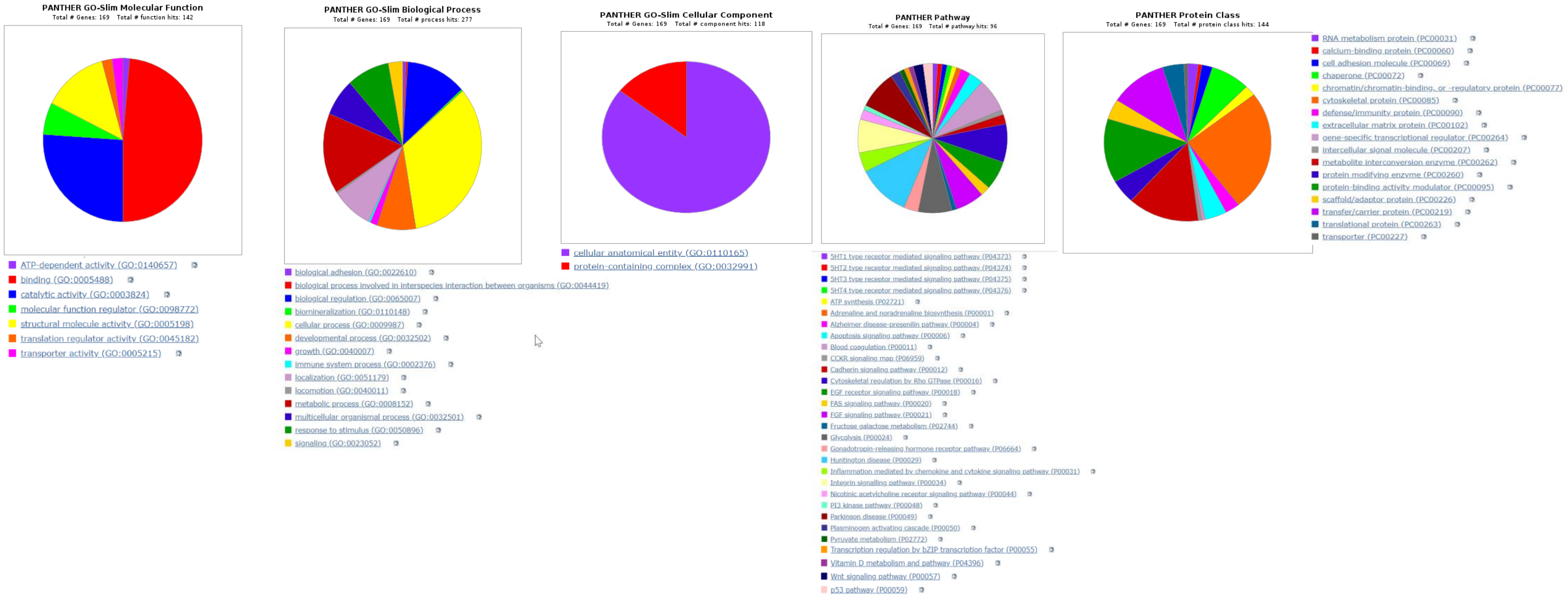

Figure S3 Pie charts showing differentially expressed secretome proteins in MDA MB-231 after treatment with NVA-IT, involved in pathways, biological processes, cellular components, and molecular functions. The values are represented as mean± SEM(n=3).

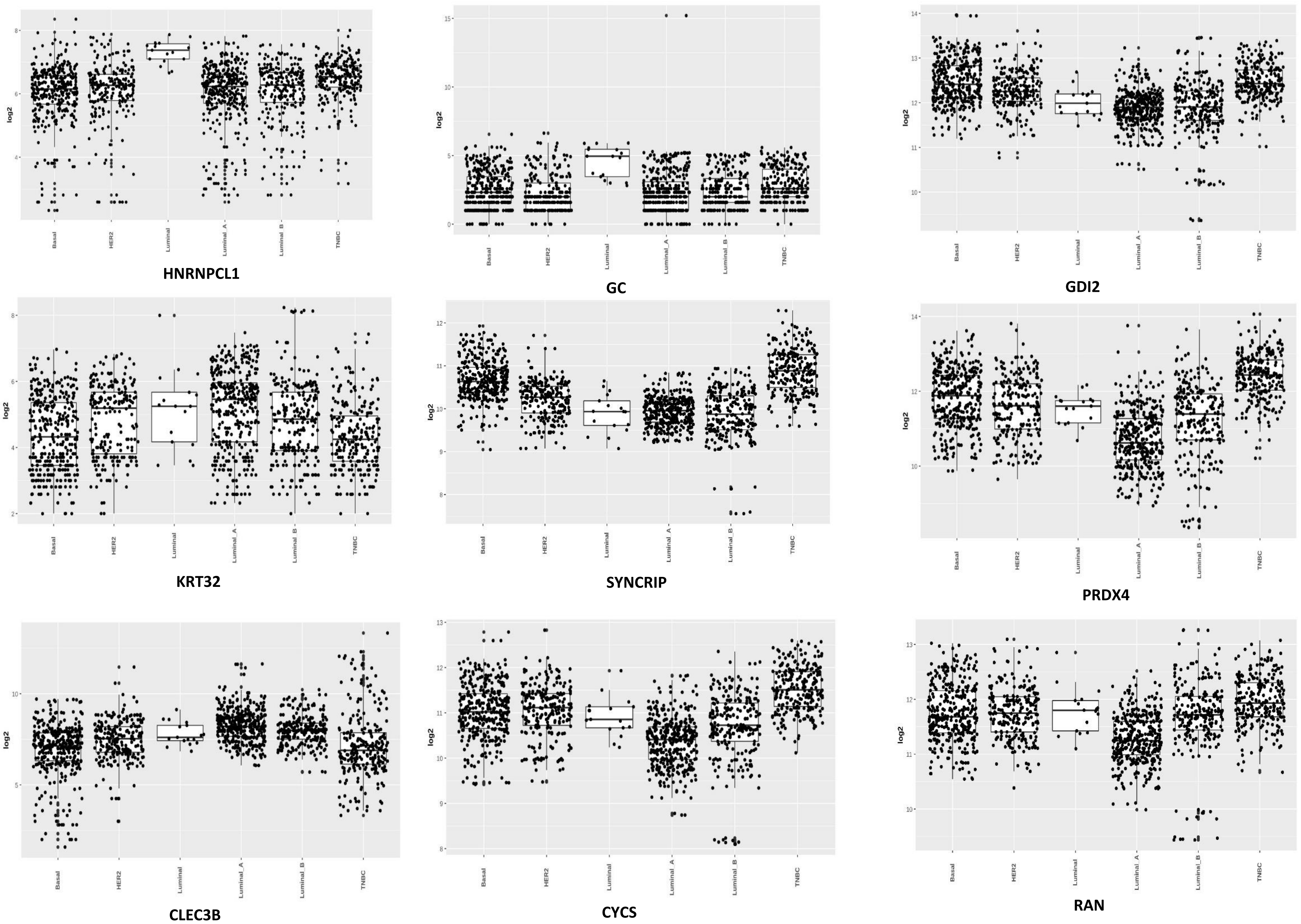

Figure S4: Expression analysis of selected proteins in different subtypes of breast cancer patients (GENT2 tool).

Table S1 Differentially expressed proteins in MCF-7 cell secretome after NVA-IT treatment.

**Protein Description**	**Gene symbol**	**Abundance Ratio (log2): (MCF-7 treatment 2) / (Control MCF-7)**
Elongation factor 1-alpha 2	EEF1A2	4.13
Histone H2B type F-S	H2BFS; LOC102724334	4.56
Histone H2B type 1-O	HIST1H2BO	4.56
Histone H2B type 1-J	HIST1H2BJ	4.56
Histone H2B type 2-F	HIST2H2BF	4.56
Histone H2B type 1-H	HIST1H2BH	4.56
Histone H2B type 1-B	HIST1H2BB	4.56
Histone H2B type 1-A	HIST1H2BA	4.56
Histone H2B type 1-C/E/F/G/I	HIST1H2BC/E/F/G/I	4.56
Histone H2B type 1-L	HIST1H2BL	4.56
Histone H2B type 2-E	HIST2H2BE	4.56
Histone H2B type 3-B	HIST3H2BB	4.56
Histone H2B type 1-M	HIST1H2BM	4.56
Histone H2B type 1-N	HIST1H2BN	4.56
Histone H2B type 1-K	HIST1H2BK	4.56
Histone H2B type 1-D	HIST1H2BD	4.56
Elongation factor 1-alpha 1	EEF1A1	4.64
Putative elongation factor 1-alpha-like 3	EEF1A1P5	4.64
Cytochrome c	CYCS	5.19
Heterogeneous nuclear ribonucleoprotein D0	HNRNPD	5.32
Rab GDP dissociation inhibitor alpha	GDI1	5.47
Zinc finger protein 618	ZNF618	5.82
Histone H2A type 2-B	HIST2H2AB	5.85

Histone H2AX	H2AFX	5.85
Heterogeneous nuclear ribonucleoprotein C-like 2	HNRNPCP5; LOC440563; HNRNPCL2	6.01
Heterogeneous nuclear ribonucleoprotein C-like 4	LOC101060301; HNRNPCL4; LOC649330; HNRNPCL3	6.01
Heterogeneous nuclear ribonucleoproteins C1/C2	HNRNPC	6.01
Heterogeneous nuclear ribonucleoprotein C-like 1	HNRNPCL1	6.01
Histone H4	HIST1H4A/F/D/J/C/H/E/I/B/K/L/ HIST2H4A-B/ HIST4H4	6.01
Pantetheinase	VNN1	6.02
Rab GDP dissociation inhibitor beta	GDI2	6.08
Histone H2A type 1-D	HIST1H2AD	6.23
Histone H2A type 1-H	HIST1H2AH	6.23
Histone H2A type 3	HIST3H2A	6.23
Histone H2A type 1-B/E	HIST1H2AB; HIST1H2AE	6.23
Histone H2A.J	H2AFJ	6.23
Histone H2A type 1-J	HIST1H2AJ; HIST1H2AK	6.23
Histone H2A type 1	HIST1H2AG; HIST1H2AM; HIST1H2AI; HIST1H2AK; HIST1H2AL	6.23
Histone H2A type 1-C	HIST1H2AC	6.23
Alpha-fetoprotein	AFP	6.52
Pigment epithelium-derived factor	SERPINF1	6.72
Complement C3	C3	6.96
Inter-alpha-trypsin inhibitor heavy chain H3	ITIH3	7.00
Keratin, type II cytoskeletal 1	KRT1	7.43
Actin, cytoplasmic 2	ACTG1	7.50
Actin, cytoplasmic 1	ACTB	7.51
Histone H2A type 2-A	HIST2H2AA4; HIST2H2AA3	7.56
Histone H2A type 2-C	HIST2H2AC	7.56
Tubulin alpha-8 chain	TUBA8; EHHADH	7.74
Putative tubulin-like protein alpha-4B	TUBA4B	7.74

Gelsolin	GSN	8.02
Inter-alpha-trypsin inhibitor heavy chain H2	ITIH2	8.20
Tubulin alpha-4A chain	TUBA4A	8.53
Histone H2A.V	H2AFV	8.54
Histone H2A type 1-A	HIST1H2AA	8.54
Histone H2A.Z	H2AFZ	8.54
Synaptic vesicle membrane protein VAT-1 homolog	VAT1	8.64
Trypsin-1	PRSS1	8.72
Keratin, type II cytoskeletal 2 epidermal	KRT2	8.81
Thrombospondin-1	THBS1	8.89
Tubulin alpha-1C chain	TUBA1C	8.97
Tubulin alpha-1B chain	TUBA1B	9.17
Vitamin D-binding protein	GC	9.40
Tubulin beta chain	TUBB	10.63
Nucleoside diphosphate kinase B	NME2; NME1-NME2	10.74
Peptidyl-prolyl cis-trans isomerase A	PPIA	11.26
Nucleoside diphosphate kinase A	NME1	11.30
Antithrombin-III	SERPINC1	11.92
Serum albumin	ALB	13.30
Lactotransferrin	LTF	14.30

TableS2 Differentially expressed proteins in MDA MB-231 cell secretome after NVA-IT treatment.

**Protein Description**	**Gene symbol**	**Abundance Ratio (log2): (MCF-7 treatment 2) / (Control MCF-7)**
Keratin, type II cytoskeletal 1b	KRT77	-2.17
Keratin, type I cytoskeletal 16	KRT16	-2.10
Keratin, type I cytoskeletal 9	KRT9	-1.82
Keratin, type II cytoskeletal 1	KRT1	-1.79
Synaptic vesicle membrane protein VAT-1 homolog	VAT1	-1.73
Complement component C7	C7	-1.64
Beta-2-glycoprotein 1	APOH	-1.42
Keratin, type I cytoskeletal 10	KRT10	-1.42
SPARC	SPARC	-1.40
Keratin, type II cytoskeletal 79	KRT79	-1.39
SPARC-like protein 1	SPARCL1	-1.37
Insulin-like growth factor-binding protein 2	IGFBP2	-1.36
Alpha-2-HS-glycoprotein	AHSG	-1.25
Apolipoprotein C-III	APOC3	-1.24
Collagen alpha-1(I) chain	COL1A1	-1.23
Ubiquitin-associated domain-containing protein 2	UBAC2	-1.17
Pantetheinase	VNN1	-1.15
Keratin, type I cytoskeletal 14	KRT14	-1.12
Keratin, type I cytoskeletal 15	KRT15	-1.12
Keratin, type I cytoskeletal 17	KRT17	-1.12
Keratin, type I cytoskeletal 19	KRT19	-1.12

Hepatocyte growth factor activator	HGFAC	-1.11
Lactotransferrin	LTF	-1.08
Fibromodulin	FMOD	-1.06
Pigment epithelium-derived factor	SERPINF1	-1.04
Antithrombin-III	SERPINC1	-1.00
Tetranectin	CLEC3B	-0.95
Heparin cofactor 2	SERPIND1	-0.93
Collagen alpha-1(VI) chain	COL6A1	-0.93
Serum albumin	ALB	-0.92
Inter-alpha-trypsin inhibitor heavy chain H2	ITIH2	-0.90
Complement factor I	CFI	-0.88
Prothrombin	F2	-0.88
40S ribosomal protein S5	RPS5	-0.85
Fibronectin	FN1	-0.83
Cartilage oligomeric matrix protein	COMP	-0.81
Kininogen-1	KNG1	-0.78
Alpha-2-macroglobulin	A2M	-0.78
Fibulin-1	FBLN1	-0.77
Zinc finger protein 618	ZNF618	-0.74
Keratin, type II cytoskeletal 2 epidermal	KRT2	-0.74
Complement C4-B	C4B; C4B_2; LOC100293534	-0.72
Adipocyte plasma membrane-associated protein	APMAP	-0.69
Transmembrane protein 94	KIAA0195; TMEM94	-0.67
Phenylalanine--tRNA ligase, mitochondrial	FARS2	-0.66
Apolipoprotein A-I	APOA1	-0.63
Alpha-amylase 1	AMY1A; AMY1C; AMY1B	-0.62
Pancreatic alpha-amylase	AMY2A	-0.62

Alpha-amylase 2B	AMY2B	-0.62
Vitamin D-binding protein	GC	-0.61
Gelsolin	GSN	-0.61
Vitronectin	VTN	-0.60
Complement C5	C5	-0.59
GTP-binding nuclear protein Ran	RAN	-0.59
Prelamin-A/C	LMNA	-0.59
Complement C4-A	C4A; C4B	-0.57
Inter-alpha-trypsin inhibitor heavy chain H1	ITIH1	-0.55
Alpha-fetoprotein	AFP	-0.55
Phospholipid transfer protein	PLTP	-0.54
Complement factor B	CFB	-0.52
Retinol-binding protein 4	RBP4	-0.52
Pregnancy zone protein	PZP	-0.51
Complement component C9	C9	-0.51
Hemoglobin subunit beta	HBB	-0.49
Hemoglobin subunit delta	HBD	-0.49
Inter-alpha-trypsin inhibitor heavy chain H3	ITIH3	-0.49
Hemoglobin subunit epsilon	HBE1	-0.49
Hemoglobin subunit gamma-1	HBG1	-0.49
Hemoglobin subunit gamma-2	HBG2	-0.49
Apolipoprotein B-100	APOB	-0.48
Thrombospondin-1	THBS1	-0.46
Keratin, type I cytoskeletal 24	KRT24	-0.43
Thyroxine-binding globulin	SERPINA7	-0.42
EGF-containing fibulin-like extracellular matrix protein 1	EFEMP1	-0.41
Lumican	LUM	-0.38
Carboxypeptidase B2	CPB2	-0.34

Carboxypeptidase N catalytic chain	CPN1	-0.27
Complement C3	C3	-0.27
Apolipoprotein E	APOE	-0.25
Rab GDP dissociation inhibitor alpha	GDI1	-0.19
Rab GDP dissociation inhibitor beta	GDI2	-0.19
Heterogeneous nuclear ribonucleoprotein Q	SYNCRIP	-0.15
Protein Z-dependent protease inhibitor	SERPINA10	-0.10
Cytochrome c	CYCS	-0.02
Peroxiredoxin-4	PRDX4	-0.01
Periostin	POSTN	0.05
Trypsin-1	PRSS1	0.13
Actin, cytoplasmic 1	ACTB	0.17
Actin, cytoplasmic 2	ACTG1	0.17
L-lactate dehydrogenase A-like 6A	LDHAL6A	0.18
L-lactate dehydrogenase B chain	LDHB	0.18
Fructose-bisphosphate aldolase A	ALDOA	0.42
Elongation factor 1-alpha 2	EEF1A2	0.43
Heat shock protein HSP 90-alpha A2	HSP90AA2; HSP90AA2P	0.51
Putative heat shock protein HSP 90-beta 2	HSP90AB2P	0.51
Hemoglobin subunit alpha	HBA2; HBA1	0.52
40S ribosomal protein S13	RPS13	0.53
Elongation factor 1-alpha 1	EEF1A1	0.74
Putative elongation factor 1-alpha-like 3	EEF1A1P5	0.74
14-3-3 protein sigma	SFN	0.76
14-3-3 protein beta/alpha	YWHAB	0.76
14-3-3 protein epsilon	YWHAE	0.76

14-3-3 protein gamma	YWHAG	0.76
14-3-3 protein theta	YWHAQ	0.76
14-3-3 protein zeta/delta	YWHAZ	0.76
L-lactate dehydrogenase A chain	LDHA	0.77
Tubulin beta-1 chain	TUBB1	0.94
Putative tubulin-like protein alpha-4B	TUBA4B	0.95
Tubulin alpha-8 chain	TUBA8; EHHADH	0.95
Heat shock protein HSP 90-alpha	HSP90AA1	0.95
Ubiquitin-40S ribosomal protein S27a	RPS27A	0.96
Ubiquitin-60S ribosomal protein L40	UBA52	0.96
Polyubiquitin-B	UBB	0.96
Polyubiquitin-C	UBC	0.96
Keratin, type II cytoskeletal 4	KRT4	1.07
Keratin, type II cytoskeletal 2 oral	KRT76	1.07
Alpha-enolase	ENO1	1.17
Histone H2AX	H2AFX	1.20
Histone H2A type 2-B	HIST2H2AB	1.20
Hemoglobin subunit zeta	HBZ	1.22
Histone H2A.V	H2AFV	1.28
Histone H2A.Z	H2AFZ	1.28
Histone H2A type 1-A	HIST1H2AA	1.28
Heat shock-related 70 kDa protein 2	HSPA2	1.29
Tubulin alpha-4A chain	TUBA4A	1.39
Tubulin beta-8 chain-like protein LOC260334		1.45
Tubulin beta-6 chain	TUBB6	1.45
Tubulin beta-8 chain	TUBB8	1.45
Phosphoglycerate mutase 2	PGAM2	1.50
Tubulin alpha-1C chain	TUBA1C	1.52

Heterogeneous nuclear ribonucleoprotein K	HNRNPK	1.59
Glyceraldehyde-3-phosphate dehydrogenase, testis-specific	GAPDHS	1.61
Heat shock protein HSP 90-beta	HSP90AB1	1.65
Tubulin alpha-1B chain	TUBA1B	1.69
Gamma-enolase	ENO2	1.73
Alpha-2-antiplasmin	SERPINF2	1.75
Histone H2B type F-S	H2BFS; LOC102724334	1.79
Histone H2B type 1-A	HIST1H2BA	1.79
Histone H2B type 1-B	HIST1H2BB	1.79
Histone H2B type 1-D	HIST1H2BD	1.79
Histone H2B type 1-H	HIST1H2BH	1.79
Histone H2B type 1-C/E/F/G/I	HIST1H2BC/E/F/G/I	1.79
Histone H2B type 1-J	HIST1H2BJ	1.79
Histone H2B type 1-K	HIST1H2BK	1.79
Histone H2B type 1-L	HIST1H2BL	1.79
Histone H2B type 1-M	HIST1H2BM	1.79
Histone H2B type 1-N	HIST1H2BN	1.79
Histone H2B type 1-O	HIST1H2BO	1.79
Histone H2B type 2-E	HIST2H2BE	1.79
Histone H2B type 2-F	HIST2H2BF	1.79
Histone H2B type 3-B	HIST3H2BB	1.79
Histone H2A.J	H2AFJ	1.82
Histone H2A type 1-B/E	HIST1H2AB; HIST1H2AE	1.82
Histone H2A type 1-C	HIST1H2AC	1.82
Histone H2A type 1-D	HIST1H2AD	1.82
Histone H2A type 1	HIST1H2AG/M/I/K/L	1.82
Histone H2A type 1-H	HIST1H2AH	1.82
Histone H2A type 1-J	HIST1H2AJ; HIST1H2AK	1.82
Histone H2A type 3	HIST3H2A	1.82

Endoplasmin	HSP90B1	1.86
Heat shock protein 75 kDa, mitochondrial	TRAP1	1.88
Beta-enolase	ENO3	1.88
Histone H2A type 2-A	HIST2H2AA4; HIST2H2AA3	1.89
Histone H2A type 2-C	HIST2H2AC	1.89
Heat shock cognate 71 kDa protein	HSPA8	1.91
Glyceraldehyde-3-phosphate dehydrogenase	GAPDH	1.94
Probable phosphoglycerate mutase 4	PGAM4	1.99
Tubulin beta-2A chain	TUBB2A	2.04
Tubulin beta-2B chain	TUBB2B	2.04
Putative heat shock protein HSP 90-beta 4	HSP90AB4P	2.07
Putative heat shock protein HSP 90-beta-3	HSP90AB3P	2.07
Phosphoglycerate mutase 1	PGAM1; LOC643576	2.09
Tubulin beta chain	TUBB	2.17
Keratin, type I cytoskeletal 18	KRT18	2.18
Keratin, type I cuticular Ha1	KRT31	2.18
Keratin, type I cuticular Ha2	KRT32	2.18
Keratin, type I cuticular Ha3-II	KRT33B	2.18
Keratin, type I cuticular Ha5	KRT35	2.18
Keratin, type I cuticular Ha6	KRT36	2.18
Keratin, type I cuticular Ha7	KRT37	2.18
Keratin, type I cuticular Ha8	KRT38	2.18
Keratin, type I cytoskeletal 28	KRT28	2.18
Alpha-actinin-1	ACTN1	2.20
Alpha-actinin-4	ACTN4	2.20
Keratin, type I cytoskeletal 25	KRT25	2.30
Keratin, type I cytoskeletal 26	KRT26	2.30

Keratin, type I cytoskeletal 27	KRT27	2.30
L-lactate dehydrogenase C chain	LDHC	2.36
Histone H4	HIST1H4A/F/D/J/C/H/E/I/B/K/L/ HIST2H4A-B/ HIST4H4	2.67
Pyruvate kinase PKM	PKM	2.79
Tubulin alpha-1A chain	TUBA1A	3.12

Table S3 Common differentially expressed proteins in both cell lines (MCF-7 & MDA MB- 231) altered after NVA-IT treatment.

S.No.	Gene symbol	Expression in Tumor VS Normal breast tissue (GENT2)	Expression after NVA-IT treatment in MDA MB231	Expression after NVA-IT treatment in MCF-7
1	GDI1	higher	downregulated	upregulated
2	HIST1H2AD	lower	upregulated	upregulated
3	TUBA4A	lower	upregulated	upregulated
4	H2BFS; LOC102724334	lower	upregulated	upregulated
5	H2AFJ	lower	upregulated	upregulated
6	TUBA4B	lower	upregulated	upregulated
7	HIST1H2BL	lower	upregulated	upregulated
8	SERPINF1	lower	upregulated	upregulated
9	LTF	lower	upregulated	upregulated
10	HIST1H2BA	lower	upregulated	upregulated
11	EEF1A1	lower	upregulated	upregulated
12	PRSS1	lower	upregulated	upregulated
13	TUBA8; EHHADH	lower	upregulated	upregulated
14	VNN1	lower	upregulated	upregulated
15	KRT2	lower	downregulated	upregulated
16	CYCS	higher	downregulated	upregulated
17	C3	lower	downregulated	upregulated
18	GDI2	higher	downregulated	upregulated
19	GC	lower	downregulated	upregulated
20	AFP	lower	downregulated	upregulated
21	KRT1	lower	downregulated	upregulated
22	ITIH2	lower	downregulated	upregulated
23	THBS1	higher	downregulated	upregulated
24	VAT1	lower	downregulated	upregulated
25	GSN	lower	downregulated	upregulated

26	ALB	lower	downregulated	upregulated
27	ITIH3	higher	downregulated	upregulated
28	ZNF618	higher	downregulated	upregulated
